# Metabolic Program of Regulatory B Lymphocytes and Influence in the Control of Malignant and Autoimmune Situations

**DOI:** 10.3389/fimmu.2021.735463

**Published:** 2021-09-28

**Authors:** Cristian Iperi, Anne Bordron, Maryvonne Dueymes, Jacques-Olivier Pers, Christophe Jamin

**Affiliations:** ^1^ LBAI, UMR1227, Univ Brest, Inserm, Brest, France; ^2^ Service d’Odontologie, CHU de Brest, Brest, France; ^3^ Laboratoire d’Immunologie et Immunothérapie, CHU de Brest, Brest, France

**Keywords:** B lymphocytes, regulatory activity, metabolism, autoimmunity, cancer, Breg cells

## Abstract

Metabolic pathways have been studied for a while in eukaryotic cells. During glycolysis, glucose enters into the cells through the Glut1 transporter to be phosphorylated and metabolized generating ATP molecules. Immune cells can use additional pathways to adapt their energetic needs. The pentose phosphate pathway, the glutaminolysis, the fatty acid oxidation and the oxidative phosphorylation generate additional metabolites to respond to the physiological requirements. Specifically, in B lymphocytes, these pathways are activated to meet energetic demands in relation to their maturation status and their functional orientation (tolerance, effector or regulatory activities). These metabolic programs are differentially involved depending on the receptors and the co-activation molecules stimulated. Their induction may also vary according to the influence of the microenvironment, i.e. the presence of T cells, cytokines … promoting the expression of particular transcription factors that direct the energetic program and modulate the number of ATP molecule produced. The current review provides recent advances showing the underestimated influence of the metabolic pathways in the control of the B cell physiology, with a particular focus on the regulatory B cells, but also in the oncogenic and autoimmune evolution of the B cells.

## Introduction

There is now a growing body of evidence showing a complex interaction between metabolic reprogramming and immunity. Innate cells and adaptive immune T cells have been studied largely, but much little is still known about B cells. Because this now adds a new dimension to our understanding of the immune system in health but also in disease, it is important to highlight what is accepted for B lymphocytes. This review will also shed in light the importance of the metabolic processes in the development of the functional regulatory capacities of B cells and the implication in autoimmune and cancer responses.

## The Metabolic Pathway in Immune B Cells

Among the specialized cells of the adaptive immune system, B lymphocytes play a pivotal role for the detection of danger signal and the presentation of antigen to T cells to mount efficient germinal center (GC) responses (1, 2). Following activation signals subsequent to a fine dialogue with the T cells, B cells acquire effector and regulatory functions leading to the production of antibodies and the secretion of cytokines, and thus develop humoral immune responses for antigen neutralization and participate to the control of the cellular immune responses (3, 4).

Three to six metabolic pathways have been described to play important role in the survival, the proliferation and differentiation of immune cells (5) which may also contribute to the development and functions of B cells.

### The Generic Pathways

#### Glucose Metabolism in B Cells

**Figure 1:**
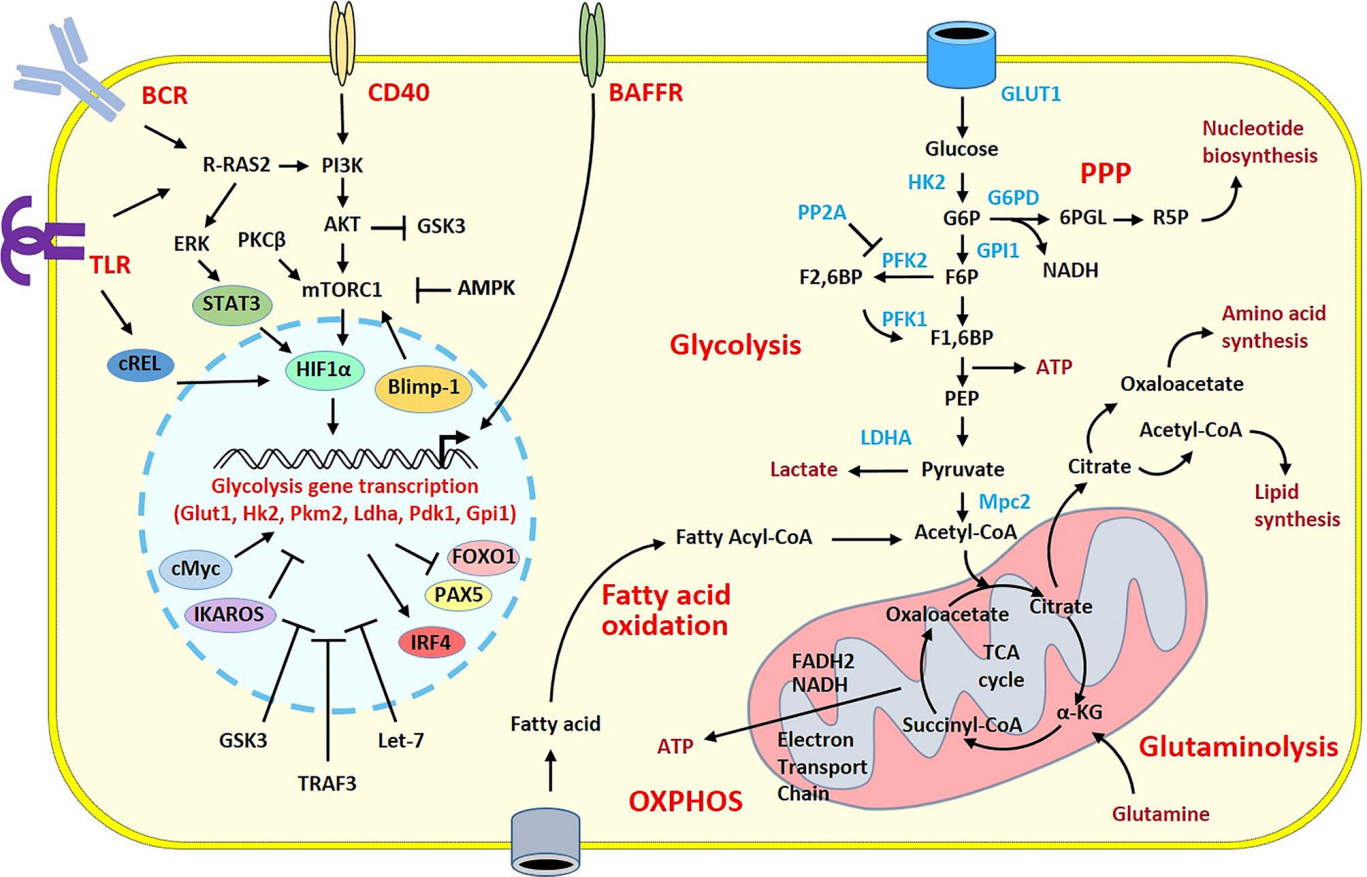
Overview of the metabolic pathways and stimulatory signals in activated B cells. Glucose has a pivotal role in the supply of energy and biomolecules. GLUT1 regulates glucose transport allowing glucose to fuel the glycolytic pathway and the TCA cycle within the mitochondria. The TCA cycle produces NADH and FADH2, which deliver electrons flux to the Electron Transport Chain for ATP generation during the oxidative phosphorylation. B cell metabolism can shunt from these pathways toward the pentose phosphate pathway (PPP) to increase nucleotide biosynthesis. In activated B cells, the high flux of glucose grants the biomolecules required for the cell expansion. Because of that, there is a large production of lactate and increased lipid synthesis. Fatty acid oxidation and glutaminolysis reactions are also increased to sustain the TCA cycle and replace the intermediates consumed in other biological processes. Stimulatory signals from the BCR and TLRs or from CD40 or BAFFR co-stimulatory molecules activate intra-cellular pathways with the recruitment of transcription factors leading to the transcription of glycolysis genes in the nucleus. The induction of the metabolic programs is under the control of numerous transcription factors, miRNA and enzymes that have positive (pointed arrow) or inhibitory (flattened arrow) effects. Only intermediates, transcription factors, enzymes and signaling molecules mentioned in the review are shown. α-KG, α-ketoglutarate; AMPK, AMP-activated protein kinase; ATP, adenosine triphosphate; BCR, B cell receptor; F1,6BP, Fructose 1,6-bisphosphate; F2,6BP, Fructose 2,6-bisphosphate; F6P, Fructose 6-phosphate; G6P, Glucose 6-phosphate; G6PD, Glucose-6-phosphate dehydrogenase; GPI1, glucose-6-phosphate isomerase; 6PGL, 6-Phosphogluconolactonase; GSK3, glycogen synthase kinase 3; HK2, Hexokinase 2; LDHA, lactate dehydrogenase A chain; OXPHOS, Oxidative phosphorylation; PEP, Phosphoenolpyruvate; PFK1, Phosphofructokinase 1; PFK2, Phosphofructokinase 2; PP2A, Protein phosphatase 2; TLR, Toll-like receptor.

The main nutrient to provide energy needs for the B cell development and maturation is glucose that is first catabolized to pyruvate during glycolysis. Hexokinase generates successively glucose 6-phosphate, fructose 6-phosphate, fructose 1,6-biphosphate and then pyruvate in order to be fermented into lactate which is then secreted. This reaction does not require oxygen, is poorly energy efficient and is finely regulated. Numerous molecules on B cells are involved in the control of glucose metabolism either positively or negatively. After B cell receptor (BCR) and CD40 stimulation of B cells, cMyc is increased and enhances glycolysis and mitochondrial biogenesis for the formation and maintenance of GCs (6). Concomitantly, the NF-κB subunit cREL is activated and increases the oxygen consumption and the glycolytic flux favoring GC B cell survival (7) (**Figure 1**).

The mammalian target of rapamycin (mTOR) complex 1 is a central controller of cell growth and proliferation. Thanks to CD40 T cell help, mTORC1 is activated in the light zone of the GC, promoting the anabolic program that sustains the B cell proliferation in the dark zone of the GC (8). mTORC1 itself is regulated by multiple signals such as growth factors, amino acids, or cellular energy. Thus, under metabolic stress, anabolic processes must be inhibited to ensure available nutrients. In this situation, the 5’ adenosine monophosphate activated protein kinase (AMPK) is one major regulator. This enzyme is activated because available energy decreases. AMPK then reduces the activity of mTORC1 to limit energy consumption (9) and promotes B cell survival under starvation. Protein Kinase C-β (PKCβ) is another important key regulator of metabolic reprogramming in B cells and B cell fate. PKCβ activates mTORC1 signaling leading to mitochondrial remodeling for plasma cell differentiation (10). Ras-related guanosine triphosphate hydrolase (GTPase) R-Ras2 plays also a pivotal role in the development of GC response and B cell expansion. R-Ras2 is essential for the mitochondrial replication and glucose metabolism to supply the energy required for the generation of effective antibody responses (11).

#### Other Metabolisms in B Cells

Glucose can be also catabolized in the pentose phosphate pathway by the glucose 6-phosphate dehydrogenase to ribose 5-phosphate used for nucleic acid synthesis (**Figure 1**). The reaction is associated with the generation of NADH which is needed for the fatty acid synthesis to provide the demands during the proliferation and the differentiation responses of the B cells (12).

Moreover, following glucose catabolization, pyruvate can be transported into the mitochondrial matrix of B cells thanks to Mitochondrial pyruvate carrier 2 (Mpc2) to be converted in acetyl-CoA which is oxidized to CO2 in the tricarboxylic acid (TCA) cycle also called Krebs or citric acid cycle. During this cascade reactions, NADH and FADH2 are generated serving as electron donors for the electron transport chain which is required for the generation of ATP molecules. This oxidative phosphorylation (OXPHOS) pathway inducing lipid metabolism is efficient in the generation of ATP and is mainly engaged during B cell proliferation. It is positively regulated by mTORC1, cMyc, PKCβ, cRel and R-Ras2, and negatively regulated by the glycogen synthase kinase 3 (GSK3) which can participate in B cell growth, proliferation and metabolic activity and the TNF receptor associated factor 3 (TRAF3) (13).

Another substrate for energy production comes from glutamine. This amino acid can be transformed into glutamate and then catabolized to α-ketoglutarate for the Krebs cycle during glutaminolysis. This pathway provides additional substrate for the generation of ATP molecules required for the B cell responses (12).

### The Provision for the Maturation

#### Naïve *Versus* Activated, Memory, and Plasma B Cells

All metabolic demands of B cells are adjusted to their functional needs, naïve resting B cells having different metabolic profile compared with stimulated activated B cells. Naïve follicular B cells entering GCs preferentially use fatty acid oxidation as an energy source over glycolysis (14). Differential metabolite needs will have further consequences, particularly in memory B cell functions, be it Ab-producing cell differentiation or re-entry into GCs for additional somatic hypermutation. Stimulation of B cell activating factor (BAFF) that provides survival signal to B cells (15) increases glycolysis and induces differentiation into Ab-producing cells (16). Upon either Toll-like receptors (TLRs), CD40 or BCR stimulation, control B cells have a balanced increase in lactate production and oxygen consumption, with proportionally increased glucose transporter 1 (Glut1) expression leading to enhance glucose uptake and mitochondrial mass (17). Consistently, the glycolytic inhibition suppresses the B cell proliferation and Ab secretion, and Glut1 defective expression reduces B cell numbers and impairs Ab production. In contrast, anergic B cells remain metabolically quiescent even after TLR stimulation, with only a modest increase in glycolysis and oxygen consumption. The Glut1-dependent metabolic reprogramming appears pivotal for the B cell proliferation and Ab production, and is a critically regulated pathway in tolerance.

Differential metabolic program clearly orients the B cell fate. High PI3K/AKT/mTOR signaling inactivates the transcription factor FOXO1 but induces the transcription factor IRF4 which drives the plasma cell differentiation. This occurs through the activation of genes encoding proteins among which GLUT1 that contribute to glycolysis. In contrast, low PI3K/AKT/mTOR signaling drives self-renewal of the B cells and leads to features of a memory or GC B cell fate inducing FOXO1 and PAX5 with decreased IRF4 level (18). This alternative differentiation arises likely through the inhibition of glycolysis (19). Blimp-1 which is a well-known transcription factor involved in plasma cell differentiation controls the size of plasma cells through the regulation of mTORC1 (20) highlighting once again the importance of precise metabolic reprogramming to adapt to the energy needs and the B cell orientation.

#### Immature *Versus* Mature B Cells

In line with the fine control of the metabolic profile, the Hypoxia-inducible factor (HIF) HIF-1α is a transcription factor recently described able to reprogram immune cell metabolism by the activation of genes encoding glucose transporters and glycolytic enzymes for the importation of glucose and its conversion into lactate. HIF-1 expression is essential for B cell development as its activity varies from high into human and murine bone marrow pro-B and pre-B cells to low into the immature B cell stage. Genetic activation of HIF-1α in murine B cells lowered surface BCR, CD19 and BAFF receptor and increased expression of pro-apoptotic BIM molecule. These modifications are associated with reduced repertoire diversity, decreased BCR editing and lead to developmental arrest of immature B cells. The HIF dynamic suppression and the associated decreased BIM expression are thus required for normal B cell development (21). Furthermore, by shifting from oxidative metabolism to glycolytic metabolism HIF-1α also regulates proliferation and function of mature B cells. HIF-1α is induced after stimulation of the BCR through the ERK-STAT3 pathway and by stimulation of TLR *via* the NF-κB pathway (22). The HIF-1α-associated enhanced glucose transport activity correlates with an induced mRNAs expression of Glut1, Hk2, pyruvate kinase M2 (Pkm2), lactate dehydrogenase A (Ldha), phosphoinositide-dependent kinase 1 (Pdk1), and glucose-6-phosphate isomerase 1 (Gpi1), all enzymes involved in glycolysis. During proliferation, lactate production increases together with consumption of essential amino acids. The ensuing plasma cell differentiation and Ig secretion are linked with alanine and glutamate production, amino acids consumption, and increased production of lactate and 5’-methylthioadenosine (MTA), glutamine being used as carbon and energy sources (23).

#### Antigenic Influence for Antibody Production

To go further in the complexity of the metabolic reprogramming, pathways will also differ depending on the antigenic stimulation and the subsets of B cells activated. During the T cell-independent (TI) short-lived plasma cell (SLPC) production from marginal zone (MZ) B cells, glycolysis is involved with mTOR activating transmembrane activator and CAML interactor (TACI) receptor pathway *via* MyD88. This intracellular signaling induces proliferation of MZ B cells and genetic recombination for the SLPC differentiation (24). The TI antigen-induced IgM antibody production is regulated by different pathways. Specifically, the acquisition and utilization of key nutrients, including glucose and glutamine, are under the control of the Let-7 miRNAs members. They inhibit the B cell activation due to suppression of glucose and glutamine utilization, through a mechanism regulating c-Myc to directly target the hexokinase Hk2 and repress the glutamine transporter Slc1a5 as well as a key degradation enzyme glutaminase (Gls). Restricting the availability of necessary nutrients alters the TI IgM production without any effect on the OXPHOS pathway (25).

In contrast to what is observed in SLPC, during the T cell-dependent (TD) class-switching and long-lived plasma cell (LLPC) production, nutrient and glucose uptake are elevated, and provide survival and energy metabolism for antibody-secreting activities (26). Throughout the development of the TD response, glycolysis is not affected in stimulated B cells, but the OXPHOS is increased, and the TCA cycle and the nucleotide biosynthesis are also elevated to respond to the metabolic demands of the B cell growth and differentiation (27). There is a positive effect of glucose uptake and catabolism on plasma cell longevity and function. LLPCs take higher levels of glucose and glutamine than do SLPCs under homeostatic conditions. Furthermore, nutrients that can be provided through autophagy are also higher in LLPCs to be catabolized and directed to mitochondria for ATP generation. These metabolites can be used in synthesis and glycosylation of antibodies. It should be noted that while endoplasmic reticulum stress is equivalent between plasma cell subsets, SLPCs degrade antibody molecules more than LLPCs. Consequently, despite equivalent rates of protein and antibody synthesis, LLPCs secrete more antibody molecules than SLPCs. Then, under limiting nutrient availability and low ATP generation, LLPCs increases their basal respiratory capacity to compensate, in contrast to SLPCs that are unable to mobilize this function and therefore initiate programmed cell death pathways (28).

L‐glutamine is known for a while to be essential for both proliferation, plasma cell differentiation and Ab production of human B cells (29). Tryptophan is another essential amino acid with significant role in sustaining immune function. Its catabolism is under the control of indoleamine-2,3-dioxygenase (IDO), using the metabolic-stress sensing protein kinase GCN2 as a primary downstream effector. However, the role of IDO is still complex to maintain proper B cell responses. IDO-1 can be associated with an immunosuppressive role whilst IDO-2 is rather associated with pro-inflammatory responses (30). Interestingly, activation of GCN2 reduces anti-DNA autoantibodies and protects lupus-prone mice from disease. This indicates that GCN2 is another additional metabolic enzyme in B cells playing a key role in regulating the tolerogenic response to apoptotic cells and limiting autoimmunity (31).

Overall, the metabolic pathways of glucose, fatty acid and amino acids appear remarkably interconnected and can be finely co-regulated to influence the B cell behavior. As an example, mTOR regulates both glycolysis and fatty acid synthesis in activated B cells, detects amino acids and growth factors, promotes mRNA translation and lipid synthesis, all pathways oriented to support B cell growth (32). The involvement of numerous physiological signaling highlights the complex interplay between cell surface receptors and the different metabolic pathways to govern the B cell maturation.

## The Intra-Signalling Pathways in the Regulatory Functions

Metabolic programs are clearly involved in the B cell development from precursor to immature stage until activation for GC responses and terminal plasma cell differentiation. However, metabolic nutrients are also required for immune cell function acquisition (33) and to switch from effector immune cells to regulatory immune cells (34). While largely delineated in T lymphocytes (35), there is much less data available in B lymphocytes (36, 37).

Based on their phenotype, B cells are grouped into B1 and B2 cells which are CD11b^+^ and CD11b^-^, respectively. The majority of B cells classified into follicular (FO) B cells located in lymphoid follicles of secondary lymphoid tissues and the circulation, and MZ B cells mainly found in the spleen belongs to B2 cells. B1 cells found in the fetal liver and in peritoneal cavity can be further divided into CD11b^+^CD5^+^ B1a cells and CD11b^+^CD5^-^ B1b cells (38).

Regulatory B (Breg) lymphocytes have been described for decades now in mice and in humans. It is still unclear whether Breg cells originated from specific precursors or appeared following stimulation of various populations from B1 and/or B2 cells. The abundant literature strongly suggests that they correspond to diverse subpopulations of B cells issued from different groups and/or from different maturation stages according to their phenotypes and/or activities. In mice, CD5^+^ B1a cells, CD5^+^CD1d^high^ B10 cells, CD21^high^CD23^-^ MZ cells, CD21^hi^CD23^hi^CD24^hi^CD1d^hi^ transitional type 2 MZ precursor (T2-MZP) cells, Tim1^+^ B2 cells, CD24^high^CD27^+^ memory cells, CD138^+^CD44^high^ plasmablast, or CD138^+^B200^+^ plasma cells have been identified as Bregs. Likewise, CD5^+^ Br3 cells, CD24^high^CD27^+^ B10 cells, CD24^high^CD38^high^ transitional cells, CD25^high^CD71^high^CD73^-^ Br1 cells, CD24^high^CD27^+^ memory cells, or CD24^high^CD27^int^CD38^+^ plasmablast in humans have been associated with regulatory activities (39). They participate to the control of tolerance and restrain the development of improper immune responses through direct cell-to-cell contact with targeted cells and/or through the production of cytokines such as IL-10, TGFβ or IL-35 (**Table 1**). Hence, dysfunctional Bregs have been suspected to contribute to the expansion of aberrant autoimmunity (40–42), of uncontrolled inflammatory responses (43), of inappropriate tumor development (44), as well as graft rejection (45) or allergic reactions (46). Notwithstanding their important contributions in the control of various immune-mediated responses, little is known about the intracellular pathways leading to their differentiation and governing the acquisition of their functions as well as the incidence of metabolic programs (47, 48).

**Table 1:** Diverse regulatory B cell subsets.

B cell type	Species	Associated cytokines	Location
CD5^+^ B1a cells	Mouse	IL-10	Spleen and Bone marrow
CD5^+^CD1d^high^ B10 cells	Mouse	IL-10	Spleen
CD21^high^CD23^-^ Marginal Zone (MZ) cells	Mouse	IL-10	Spleen
CD21^high^CD23^high^CD24^high^CD1^high^ Transitional type 2-MZ Precursor cells	Mouse	IL-10	Spleen
Tim1^+^ B2 cells	Mouse	IL-10	Spleen and lymph nodes
CD25^high^CD69^high^ B2 cells	Mouse	TGFβ	Blood and inflammation sites
CD24^high^CD27^+^ memory cells	Mouse	IL-10	Blood
CD138^+^CD44^high^ plasmablasts	Mouse	IL-10	draining lymph nodes
CD138^+^B200^+^ plasma cells	Mouse	IL-10 and IL-35	Spleen
CD5^+^ Br3 cells	Human	TGFβ	Blood
CD24^high^CD27^+^ B10 cells	Human	IL-10	Blood
CD24^high^CD38^high^ transitional cells	Human	IL-10 and TGFβ	Blood and inflammation sites
CD24^high^CD27^+^ memory cells	Human	IL-10 and TGFβ	Blood and inflammation sites
CD25^high^CD71^high^CD73^-^ Br1 cells	Human	IL-10	Blood
CD24^high^CD27^int^CD38^+^ plasmablasts	Human	IL-10	Blood

### The Activation Pathways

Generally speaking, activation of B cells is triggered through ubiquitous pathways. Syk tyrosine kinase phosphorylates the phospholipase C gamma 2 (PLCγ2) leading to the cleavage of the phosphatidylinositol-4,5-bisphosphate (PI (4,5)P2) into inositol 1,4,5-trisphosphate (IP3) and diacylglycerol (DAG). With calcium entry, binding of IP3 on its receptor on the endoplasmic reticulum induces activation of the calmodulin kinase (CaMK II) and of the calcineurin (phosphatase 2B). The phosphatase activity of calcineurin enables activation of the p38 Mitogen-activated protein kinase (MAPK) cascade for the translocation of NFAT into the nucleus to launch the B cell response (49). These pathways can be differentially activated to orient the B cell regulatory activities based on the stimulating receptor engaged.

Stimulation of the BCR can generate calcium-dependent signals responsible for the development of Breg function specifically the production of the anti-inflammatory cytokine IL-10 due to induction of the store-operated calcium (SOC) influx (50). SOC influx is triggered by depletion of calcium from the endoplasmic reticulum which is dependent on the stromal interaction molecule (STIM) 1 and STIM2 sensors. B cell-specific deletion of STIM1 and STIM2 in mice causes altered NFAT activation after BCR stimulation and subsequently decreased production of IL-10 resulting in exacerbation of autoimmune disease. These observations highlight the STIM-dependent SOC influx following BCR engagement as a critical signal for the development of effective Breg cells capable to limit autoimmunity.

TLR9 is another well-known potent activator of Breg functions (51, 52). Recently, TLR9 stimulation has been shown to induce production of IL-10 as well as the pro-inflammatory cytokine IL-6 but through partially disconnected intra-cellular pathways. IL-10 and IL-6 secretion are both dependent on the Syk and Bruton’s tyrosine kinases. However, the TLR9-induced IL-10 production and secretion by Breg cells specifically requires calcineurin pathway with increased phosphorylation of CaMKII, whilst IL-6 production is not associated with this calcium-dependent pathway (53). Nevertheless, the TLR9-dependent IL-10 production does not require the phosphatase activity of calcineurin and is negatively regulated by NFAT (54).

Aryl hydrocarbon receptor (AhR) described as an important regulator of the development and function of innate and adaptive immune cells (55) is the latest identified transcription factor involved in the control of the differentiation and function of Bregs, among which the IL-10-producing CD21^hi^CD24^hi^ Breg cells in mice and in humans. AhR retrains their differentiation into cells that contribute to inflammation, since the loss of AhR orients the cells towards inflammatory profile with diminished production of IL-10 (56). AhR-deficient mice show significant reduction in IL-10-producing Breg cells and regulatory T (Treg) cells, and display increased Th1 and Th17 cells associated with exacerbated inflammation and arthritis. Arthritic mice, similarly to rheumatoid arthritis (RA) patients who have lower butyrate levels compared to healthy controls, have a significant reduction in microbial-derived short-chain fatty acids (SCFAs) (57). A SCFA butyrate supplementation can induce two AhR ligands associated with Trp metabolism, 5-hydroxyindole-3-acetic acid and kynurenic acid, which lead to an increased production of IL-10 transcription in T2-MZP Breg cells, likely through the downstream p38 MAPK activation (58). The AhR-dependent orientation of B cells into IL-10 Breg is correlated with the inhibition of GC and plasmablast differentiation and ultimately with a reduction of the pro-arthritogenic response.

Importantly, the B cell fate also depends on the BCR signal strength. A recent study demonstrated that weak BCR engagement induces apoptosis due to the inability to activate NF-kB-driven anti-apoptotic gene expression. In contrast strong BCR engagement triggering robust calcium response promotes NF-kB-dependent survival and NFAT-, mTORC1-, and c-Myc-dependent survival and proliferative response (59). CD40 or TLR9 co-stimulation, known to be inducers of functional Bregs (60) avoid these calcium-regulated checkpoints compensating for weak BCR/calcium signals to rescue NF-kB- and mTORC1-dependent fates. These observations suggest that CD40 and TLR9 activation providing stronger stimulating signals allow Breg function to develop by bypassing stimulating NF-kB and mTORC1 pathways in B cells.

### The Metabolic Pathways

**Figure 2:**
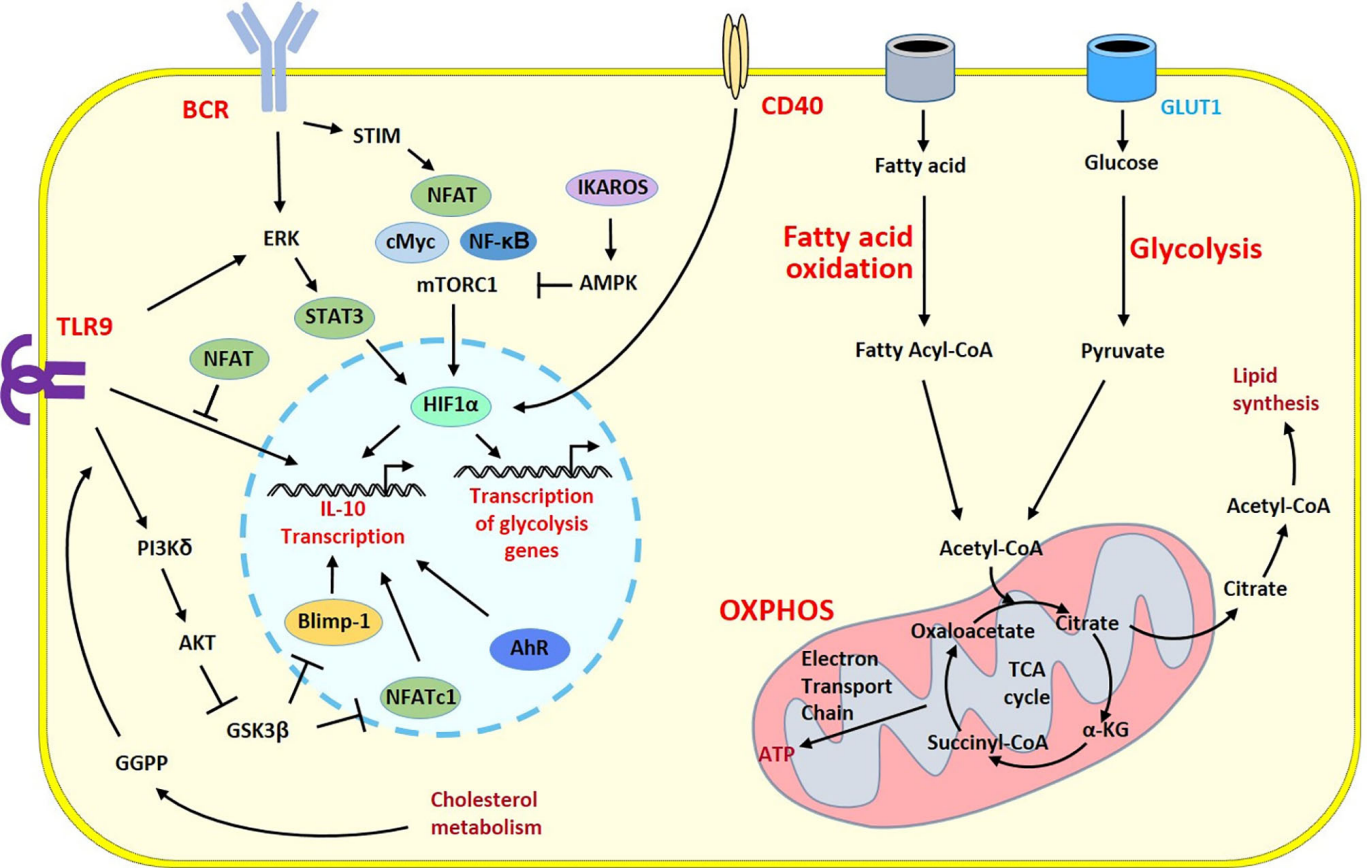
Survey of metabolic programs in Breg cells. Metabolic and regulatory programs share common signaling cascades in B cells developing regulatory properties. Activation signals from the BCR and TLR9 induce different intracellular pathways triggering HIF-1α a key player that govern transcription of the prototypic regulatory cytokine IL-10 gene and genes involved in the metabolic programs into the nucleus. The polarization into Breg cells is under the control of a number of transcription factors and enzymes having positive (pointed arrow) or inhibitory (flattened arrow) effects. Glycolysis has a central role to generate energy and biomolecules. Through the GLUT1 transporter, glucose fuels the TCA cycle within the mitochondria. During the TCA cycle, energy is generated through oxidative phosphorylation and fatty acid oxidation brings additional substrates to the TCA cycle and increases lipid synthesis. One intermediate of this pathway, the GGPP molecule produced during cholesterol metabolism induces Blimp1 another key transcription factor of the Breg program following TLR9 stimulation leading to enhanced IL-10 production. The co-stimulatory CD40 molecule can also stimulates HIF-1α to circumvent the BCR-dependent cascade and contributes to the Breg polarization. α-KG, α-ketoglutarate; AMPK, AMP-activated protein kinase; ATP, adenosine triphosphate; BCR, B cell receptor; GGPP, geranylgeranyl pyrophosphate; GSK3, glycogen synthase kinase 3; OXPHOS, Oxidative phosphorylation; STIM, stromal interaction molecule; TLR, Toll-like receptor.

Despite the high diversity of subset, little information is beginning to emerge on the behavior of metabolic pathways in Breg cells. The development of Bregs and Breg functions seem to be also closely associated with metabolic reprogramming (**Figure 2**). HIF-1α and HIF-2α transcription factors are essential for the induction of cellular response to hypoxia. Their regulatory functions have been demonstrated in macrophages and T lymphocytes showing their involvement in inflammatory and infection responses (61, 62). In B lymphocytes, TLR or BCR stimulation also induces the expression of HIF-1α, in an oxygen-independent way. The induction of the *HIF-1a* gene is mediated by the ERK-STAT3 pathway, with Ser727 phosphorylation of STAT3 (22). In HIF-1α-B cell deficient mice, a reduced number of CD5^+^CD1d^hi^ Breg cells is observed in the peritoneum with a decreased production of IL-10. The reduced Breg number is associated with lowered number of Tr1 Treg cells and intensified autoimmune manifestation. HIF-1α is important for the expansion of the IL-10 Bregs not only in the peritoneal cavity but also in the bone marrow, the spleen and the inguinal lymph nodes of the mice (22). CD5^+^CD1d^hi^ Breg cells preferentially use the glucose metabolism since they display an increased glucose transport activity compared to the CD5^-^CD1d^low^ B cells. The impaired expansion of CD5^+^CD1d^hi^ IL-10 Bregs in HIF-1α-deficient mice is associated with a decreased expression of the *Glut1, Pkm2, Hk2, Ldha, Pdk1* and *Gpi1* glycolytic enzymes in all compartments. These observations highlight the glucose metabolism as an essential mechanism for the expansion of the B1a Bregs regardless of the compartment (63). In wild-type mice, TLR or BCR activation induces the expression of HIF-1α which binds to hypoxia response elements in the *IL-10* promoter essential for the production of IL-10. By induction of glycolysis, HIF-1α also participates in the expansion of the CD5^+^CD1d^hi^ Breg cells which then recruit Treg lymphocytes for a better control of tolerance (64).

However, the development of B1 B cells and their related Breg functions are still not completely understood and the metabolic program is increasingly an underestimated safeguard. The IKAROS transcription factor encoded by the *IKZF1* gene is a multifunctional protein regulating not only hematopoietic stem cells and lymphopoiesis (65), but also coordinating self-renewal which is a main characteristics of B1 B cells (38, 66). Deletion of IKAROS in mice leads to an increase of splenic and bone marrow B1 cells with down-regulation of signaling components important for inhibiting proliferation and immunoglobulin production (67). *IKZF1* enforces a state of chronic energy deprivation, resulting in constitutive activation of the energy-stress sensor AMPK (68) and reduced mTORC1 activity (9) to limit energy consumption. Hence, IKAROS appears as a negative regulator of the development and the functions of B1 B cells through the control of the AMPK-dependent metabolic pathway.

Balance between positive and negative regulators influences the development of B1 B cells. It is also interesting to note that B1 B cells, considered as innate like B cells involved in the first line of defense against pathogens (69), are bioenergetically more active than normal B2 B cells. They not only depend on glycolysis but show higher rates of glycolysis and oxidative phosphorylation. They take up exogenous fatty acids and store lipids in droplet form. These specific characteristics suggest that B1 B cells follow distinct metabolism to adapt to their specific functional properties (70) as well as their particular localization (71). cMyc which is up-regulated in B1 B cells (72) could be a pre-determining genetic factor in the unique B1 B cell metabolic characteristics as it is a positive regulator of glycolysis and oxidative phosphorylation (16, 73), and subsequently an intrinsic factor in the orientation towards Breg capacities.

The PI3Kδ-AKT-GSK3 cascade is an alternative signaling pathway of Breg cells. This pathway drives IL-10 production under the control of cholesterol metabolism. Synthesis of the metabolic intermediate geranylgeranyl pyrophosphate (GGPP) is required to specifically drive the BLIMP1-dependent IL-10 production in all Breg subsets measured. The provision of GGPP is mandatory for the induction of the regulatory function to attenuate Th1 responses. Thus, patients with inherited gene mutations in cholesterol metabolism develop severe auto inflammatory syndrome associated with poor IL-10 production (74). Metabolic supplementation with GGPP can reverse the defect, and synthesis of this metabolic intermediate, from the Acetyl-CoA cascade prior to stimulation, activates intracellular pathways inducing the transcription factor BLIMP1, known to contribute to the plasma cell differentiation (75). Stimulation of TLR9 can activate the PI3Kδ-AKT-GSK3 cascade inducing BLIMP1, which unexpectedly promotes *IL-10* gene expression (76) driving the regulatory function of B cells (**Figure 2**).

Overall, B1 and B2 cells exhibit distinct metabolic profiles and B1a and numerous Bregs produce IL-10 through distinct metabolic pathways. These observations raise two important questions. The first is to know whether common metabolic program can be found in all Breg subpopulations or if each Breg subset develops regulatory activity through specific metabolic cascades. The second is to determine which metabolic programs are altered in Bregs in pathological situations. The consideration of the Breg subset involved and their particular environment should likely help to understand the trigger signals. Further investigations are needed.

## Malignant and Autoimmune Situations of B Cells

### The Abnormal Metabolic Program

The importance of the metabolic remodeling in malignant B cell development is now well documented. cMyc but also HIF-1α are important regulators of the glucose metabolism involved in the oncogenic transformation of the B cells (77). Moreover, cholesterol, GGPP, oxysterols and SCFAs are lipid metabolites that affect the lipid metabolism and favor the oncogenic transformation. The microenvironment factors such as hypoxia and nutrients competition affect also the B cell lipid metabolism which contribute to the development of tumor-associated B cells (78).

Furthermore, the metabolic regulation of B cells is of importance for the understanding of their role. *In‐vitro* studies have shown that glucose impairs B1 cell function in diabetes. In high concentrations, glucose reduces the differentiation of B1 cells into antibody‐producing cells and their secretion of IgM in association with decreased proliferation and increased apoptosis (79). Although not clearly understood, these observations highlight the importance of the metabolic environment for the functional orientation of the B cells and likely enough into Breg cells, and the impact that metabolic deregulation can have on autoreactive and malignant B cell development (80).

Additional molecules are involved in the control of B cell metabolism to impede the malignant transformation by the restriction of nutrients. The transcription factors PAX5 and IKAROS are not only critical for B cell development but act also as negative regulators to control aberrant B cell transformation. They enforce a state of chronic energy deprivation, by limiting the glucose uptake and the ATP levels acting as gatekeeper in B cell malignancies. *PAX5* and *IKZF1* genes are thus altered in B cell acute lymphoblastic leukemia or in BCR-ABL1-transformed pre-B cells in which glucose uptake and ATP levels are increased (68). The feedback inhibitors of the MyD88-NF-κB signaling pathway are thus impaired in malignant transformation of B cells leading to systemic inflammation due to increased glucose uptake and energy supply. Moreover, there is a constitutive low PPP activity in normal B cells and a transcriptional repression of G6PD and other key PPP enzymes by PAX5 and IKZF1 to avoid the B cell transformation. In tumor B cells, the serine/threonine-protein phosphatase 2A (PP2A) redirects glucose from glycolysis to the PPP utilization to salvage oxidative stress, reprogram the increased metabolic demand and activate the oncogenic transformation (81). B cell malignancies are clearly associated with an uncontrolled specific metabolic program. When energetic needs required for proliferation and maturation during the B cell development are no longer under control, aberrant cell growth and malignant transformation arise which might also affect Breg subsets.

Autoimmunity is another abnormal situation of particular B cell expansion or tolerance breakdown associated with modification of the metabolic profile. Systemic lupus erythematosus (SLE) B cells exhibit elevated mTORC1 activity facilitating the plasmablast expansion (82). TLR9 stimulation associated with IFN-α activate mTORC1 and produce lactate that induce the differentiation of CD27^+^IgD^+^ unswitched memory B cells into CD27^high^CD38^high^ plasmablasts (83). In contrast, TLR9 stimulation alone activates AMPK and suppress mTORC1 leading to the differentiation of the CD27^+^IgD^+^ unswitched memory B cells into CD27^-^IgD^-^ memory B cells associated with elevated cytokine production. While plasma cells mainly rely on glycolysis and memory B cells mostly depend on oxidative phosphorylation, this study indicates that aberrant mTORC1 expression in SLE contributes to increased plasmablast expansion that might also influence activities of Bregs.

Susceptibility gene that characterizes autoimmune situations could be associated with metabolic-dependent B cell dysfunctions. As a recent example in mice, increased expression of solute carrier family 15 member 4 (SLC15A4) encoded protein, an endosomal transporter regulating endosomal pH, lowered endolysosomal pH and therefore reduces the threshold for mTOR-dependent TLR7 or TLR9 activation (84). This unexpected link would facilitate an aberrant IFN type I response in human SLE patients in which SLC15A4 susceptibility gene has been observed although this has yet to be demonstrated (85). Such dysfunctions could be extended to the effectiveness of Breg activities, but this remains also to be evaluated.

### The Inappropriate Activation

Aberrant activation of metabolic programs could result from improper stimulatory signals. Deficiency of the transcription factor IKAROS, encoded by the *IKSF1* gene, is not only associated with decreased number of B cells and lack of plasma cells (86) but also with hyper-activation of TLR signaling in autoreactive B cells (87). Through the promotion of the BCR anergy and the restriction of TLR signaling, IKAROS appears to be another gatekeeper preventing autoimmunity in its property of modulating the metabolic nutrients in B cells.

Other molecules can be also involved. As mentioned earlier, BAFF and BCR signals increase glucose metabolism and glycolysis. They promote pyruvate influx into the mitochondria, which is essential for the survival of LLPC due to an increased expression of mTORC1 (88). Moreover, BAFF overexpression contributes to autoimmune disorders in mice as well as in humans (15, 89). In accordance with this differential activated cascades, inhibition of mTORC1 pathway by rapamycin treatment of Raji and Daudi human B cell lines inhibit BAFF-induced proliferation and survival by activation of PP2A and suppression of the mTOR-mediated Erk1/2 signalings (90). The generation of antibody-secreting cells is then abrogated (91). Similarly, in lupus-prone mice, B cells hyperexpressed PI3K/AKT/mTOR molecules. Rapamycin inhibit the B cell survival, proliferation and autoantibody production (90). As suggested in SLE, these observations are consistent with the idea that autoimmune B cells may be associated with mTORC1-dependent increased metabolic activity (92, 93). Thus, in a transgenic mouse model, B cells overexposed to BAFF exhibit high glycolytic phenotype and produce increased lupus-like autoantibodies (16).

TRAF3 is an adaptor molecule able to drive metabolic reprogramming supporting autoreactive B cell activation. Deficiency of TRAF3 leads to the induction of Glut1 and HK2 accompanying glucose uptake, increased anaerobic glycolysis and oxidative phosphorylation (94). Moreover, deletion of this signaling molecule in mice enhances cell survival leading to B-cell lymphoma development associated with hyperglobulinemia and enhanced TI-antibody responses (95). And as the mice aged, the pathological situation progresses into autoimmune manifestations (96). TRAF3 appears as another critical regulator of peripheral tolerance. Its defective expression results in autoimmunity through metabolic reprogramming in B cells.

Among recent molecules identified has metabolic regulators in B lymphocytes, GSK3 is instrumental for the generation and maintenance of GC B cells (97). GSK3 limits cell growth, proliferation and metabolic activity in resting B cells. But after BCR stimulation and CD40 co-activation, GSK3 regulates the B cell size, the mitochondrial biogenesis, the glycolysis and the production of ROS, to prevent apoptosis. Activation of GSK3 and mTORC1 is induced by the PI3K pathway to enhance glycolysis and respond to the novel energy needs (37). The adaptor molecules Tandem PH domain containing proteins (TAPPs) target the PI3K pathway and can regulate this metabolic program. TAPPs bind with high specificity to PI (3,4)P2. Uncoupling in TAPP Knock In (KI) mice induces B cell activation, abnormal GCs and autoimmunity. Activation of TAPP KI B cells increases GSK3 activation and increases expression of glucose transport GLUT1 and glycolysis contributing to the development of autoreactive B cells (98). Inhibition of the PI3K may reverse the TAPP-dependent increased metabolic activity, decreasing GLUT1 expression and glucose uptake, and therefore reduces the autoantibody production. These observations emphasize that GSK3 and its regulators are involved in the control of tolerance, and defective signals in autoimmune B cell expansion through metabolic reprogramming.

Interestingly, GSK3β has been recently found highly expressed in CD24^hi^CD27^+^ memory Breg cells. GSK3β specific inhibition increases their proportion and immunosuppressive function through enhanced expression of NFATc1 leading to IL-10 secretion (99). The proportions of the Breg subsets CD24^high^CD38^high^ transitional cells and CD24^high^CD27^int^CD38^+^ plasmablasts were also increased but not those of TIM1^+^ B2 cells or CD25^high^CD71^high^CD73^-^ Br1 cells. Inhibition of GSK3β thus appears as an interesting approach to activate memory Bregs of pathological situations in which inflammation is associated with defective Bregs like chronic graft-*versus* host disease. However, such strategy should be carefully planned. While CD24^hi^CD27^+^ memory Breg cells are also numerically decreased and functionally impaired in the peripheral blood of RA patients, they are increased in the synovial fluid and associated with bone destruction (100). In this case, inhibition of GSK3β resulting in enhanced Breg activity would be detrimental. Overall, future therapeutic strategy that would target metabolic pathways of Breg cells must be cautionally forecasted, however exciting it is and considered according to the particularities of each disease.

## Conclusion

It has been recently postulated that metabolic restrictions might pave the way for the elimination of malignant as well as autoreactive B cells. This may happen through ATP deprivation and oxidative damage called hyperactivation-induced metabolic stress (101). Therapy targeting mTOR activation with rapamycin or N-acetylcysteine could be also a promising way to reduce the disease severity through the restoration of efficient functional B cells. Regulation of the fatty acid pathways, such as glucocorticoid treatment, is directly linked to reduce leptin levels through inhibition of mTOR in SLE patients. Therefore, drugs that modulate metabolic processes might ameliorate the aberrant immune responses.

Because Bregs are functional B cells that control the immune responses, they must be considered as potent therapeutic cells in autoimmunity (40), in cancer (102), but also in transplantation (103) and in allergy (104). Targeting Bregs could go through the metabolic programming (105) opening new field of treatment investigations either to enhance or to decrease their activities depending on the pathophysiological conditions.

In conclusion, metabolic networks appear more than ever as interesting targets for safe therapeutic modulation of the signaling pathways that direct the regulatory function of the B cells. Taken as a whole, deciphering the metabolic programs of normal and disease-associated B cells, and in particular Breg cells should help in the identification of drugs capable of restoring normal behavior to set up the proper orientation of dysfunctional B cells underlying malignant and autoimmune situations.

## Author Contributions

CJ was in charge of writing the manuscript. CI, AB, MD, and J-OP contributed to the text. All authors contributed to the article and approved the submitted version.

## Funding

This work was supported by the Agence Nationale de la Recherche under the “Investissement d’Avenir” program with the Reference ANR-11-LABX-0016-001 (Labex IGO). CI was funded by the Université de Brest and the Région Bretagne.

## Conflict of Interest

The authors declare that the research was conducted in the absence of any commercial or financial relationships that could be construed as a potential conflict of interest.

## Publisher’s Note

All claims expressed in this article are solely those of the authors and do not necessarily represent those of their affiliated organizations, or those of the publisher, the editors and the reviewers. Any product that may be evaluated in this article, or claim that may be made by its manufacturer, is not guaranteed or endorsed by the publisher.
